# Dietary behaviour and parental socioeconomic position among adolescents: the German Health Interview and Examination Survey for Children and Adolescents 2003–2006 (KiGGS)

**DOI:** 10.1186/s12889-015-1830-2

**Published:** 2015-05-19

**Authors:** Jonas D. Finger, Gianni Varnaccia, Thorkild Tylleskär, Thomas Lampert, Gert B. M. Mensink

**Affiliations:** Department of Epidemiology and Health Monitoring, Robert Koch Institute, Berlin, Germany; Centre for International Health, Department of Global Public Health and Primary Care, University of Bergen, Bergen, Norway

**Keywords:** Socioeconomic position, Nutrition, Physical activity, Adolescents, Germany

## Abstract

**Background:**

The positive association between parental socioeconomic position (PSEP) and health among adolescents may be partly explained by dietary behaviour. We investigated the associations between fruit intake, vegetable intake, energy-dense food intake, the Healthy Nutrition Score for Kids and Youth (HuSKY) and parental education in a nationwide, cluster-randomized sample of adolescents in Germany.

**Methods:**

The German Health Interview and Examination Survey for Children and Adolescents 2003–2006 (KiGGS) included 17,641 individuals aged 0–17 years and their parents. Complete information on relevant variables was available for 6359 individuals in the 11–17 age group. The associations between nutrition indicators and parental education were analysed separately for boys and girls, using multivariate logistic regression analysis. Odds ratios (ORs) adjusted for age, region, income, occupation, physical activity and weight status related variables, were calculated for the associations between parental education and nutrition indicators.

**Results:**

After full adjustment, higher parental education level was associated with lower energy-dense food intake – with an OR of 1.3 (95 % CI 1.0–1.7) for boys with secondary educated parents and 1.8 (1.4–2.3) for boys with tertiary educated parents compared to boys with primary educated parents; the corresponding ORs for girls were 1.2 (0.9–1.5) and 1.6 (1.2–2.2). Higher parental education was associated with higher fruit intake – with an OR of 1.3 (1.0–1.7) for boys with secondary educated parents and 2.0 (1.5–2.7) for boys with tertiary educated parents compared to boys with primary educated parents; the corresponding ORs for girls were 1.0 (0.8–1.4) and 1.5 (1.0–2.1). Among boys and girls with tertiary educated parents compared to those with primary educated parents an OR of 1.3 (CI boys: 1.0–1.7, CI girls: 1.0–1.6) was observed for high vegetable intake. Among boys with tertiary educated parents compared to boys with primary educated parents an OR of 1.6 (1.2–2.2) was observed for a high HuSKY; the corresponding OR for girls was 1.5 (1.1–1.9).

**Conclusions:**

A high PSEP is associated with consumption of less energy-dense food, more fruits and vegetables and more favourable overall dietary behaviour. Preferably school-based interventions are needed to promote healthy dietary behaviour among German adolescents and a special effort is needed to reach adolescents from low-PSEP families.

**Electronic supplementary material:**

The online version of this article (doi:10.1186/s12889-015-1830-2) contains supplementary material, which is available to authorized users.

## Background

Dietary behaviour is a major determinant of health [1]. Studies, reviewed in Vlismas et al., suggest that the positive association between socioeconomic position (SEP) and health may be partly explained by dietary behaviour [2]. If SEP differences in dietary behaviour are investigated, physical activity patterns should also be considered, since energy balance is based on energy intake and total energy expenditure (TEE) [3]. A previous analysis suggests that German adults with a low level of education have higher levels of TEE than those with a high level of education, because they have higher levels of work-related physical activity, although they are less active in leisure time [4]. The observed SEP differences in physical activity level among German adolescents were smaller than those observed among adults [5]. However, it was shown that adolescents with less educated parents have higher media consumption and lower levels of TEE than those with highly educated parents [5]. This contrasts with the patterns that were observed among adults and it was hypothesized that the higher media use among adolescents from low-educated families might be linked to familial leisure time activity level. The families of manual workers (mainly lower educated) may stay at home in the evenings and weekends more often, whereas in sedentary worker families (mainly higher educated) the parents may be more active with their children in leisure time to balance their lack of activity at work [5]. Furthermore, it was shown that the observed higher energy-dense food intake among adults with low SEP may be partly explained by their higher level of physical work activity [6]. Review studies indicate that, similar to their parents, adolescents with low SEP background show more unfavourable dietary behaviours – high energy-dense food intake and low fruit and vegetable intake – than adolescents with high SEP background [7, 8]. However, the associations between SEP and dietary behaviours among adolescents have to date not been studied in a large, population-based sample in Germany. Also it is unknown which role physical activity patterns may play for the association between SEP and energy-dense food intake among adolescents. When investigating these associations body mass index (BMI) and perceived weight status need to be considered also, since being overweight is linked to SEP [9] and it is a main motivation to change dietary behaviour. Among adolescents, SEP is usually obtained using the levels of parental education, parental occupation and household income [10], as indicators of parental socioeconomic position (PSEP).

In light of the recent obesity epidemic among adolescents and its unequal distribution by social status – about 18 % of German adolescents aged 11 to 17 years have overweight or obesity and those with low PSEP are the most affected [11], there is an urgent public health concern to examine the relation between PSEP and dietary behaviour in this age group.

This study aimed at investigating the associations between PSEP indicators (education, occupation, income) and dietary behaviour (fruit intake, vegetable intake, energy-dense food intake and overall healthy diet) among adolescents. Furthermore, it aimed to examine the role of variables which may influence these associations, such as physical activity and weight status related variables.

## Methods

The German Health Interview and Examination Survey for Children and Adolescents (KiGGS) is a population based, cross-sectional survey, with data collected from May 2003 until May 2006. The overall response rate was 66.6 % [12]. Participants were randomly selected from local population registries in 167 sample points which were randomly distributed according to the structure of the Federal States and municipalities of Germany. The methods have been described in detail elsewhere [12].

The survey was approved by the Charité Universitätsmedizin Berlin ethics committee [13]. Participants aged 11 to 17 years – hereafter referred to as ‘adolescents’ – gave informed written or oral assent (at 14 years and above) and their parents signed a written informed consent. Participants underwent physical examination and completed a self-administered health questionnaire and a food frequency questionnaire (FFQ) [14]. Body weight and height was measured in a standardized way. Parents of the participants were also invited to give information for the survey. After exclusion of individuals with missing data, the final sample comprised 6359 participants. The item-response rate for the FFQ was 95.3 %. According to a previous response analysis, on average the parents of respondents had a higher education level than the parents of the non-respondents [5].

### Parental socioeconomic position

*‘Parental education’* was obtained by asking the parents two questions about the highest school and vocational training certificate attained by the mother and the father of the participant. A categorical education variable was generated for the mother and the father separately by applying a revised version of the ‘Comparative Analysis of Social Mobility in Industrial Nations’ (CASMIN) classification of education for Germany [15]. CASMIN distinguishes between primary (elementary), secondary and tertiary (higher) levels of education (see Additional file 1: Figure S1) by considering the length of educational experience, the intellectual abilities required and the value of the educational certificate achieved [16]. The highest education level of either parent was used to define the ‘parental education’ level.

*‘Parental occupation’* for each parent was assessed with a question about the ‘current or last professional position’. A categorical occupation status variable was constructed according to a revised version of the ‘Occupational Prestige in Comparative Perspective’ approach for Germany to categorizing respondents into three groups of occupation status (low, middle, high) [17]. The highest occupation status of either parent was used to define ‘parental occupation’ status.

*‘Household income’* was assessed based on two questions about the households’ approximate monthly net income and the number of persons living permanently in the household. A household net equivalent income variable was constructed by assigning need-specific weights to the household members (Organisation for Economic Co-operation and Development, OECD-modified scale: head of household = 1, additional adult household members = 0.5, children = 0.3 [18]), calculating the household size, and dividing the monthly net income by the household size. A categorical variable was created by calculating tertiles of the ‘household income’ variable (low, middle, high).

### Dietary behaviour

The ‘*Healthy Nutrition Score for Children and Youth*’ (HuSKY) is an overall healthy nutrition index developed by Kleiser et al. and has been described in detail elsewhere [19]. In short, the national ‘optimized mixed diet’ (OMD) recommendations for German children and adolescents [20] were related to the ‘KiGGS – FFQ’ information. The dietary intakes of eleven food groups (beverages, vegetable, fruit, fish, bread/cereals, pasta/rice/potatoes, milk/dairy products, eggs, meat/sausage, fats and sweets/fatty snacks/soft drinks) were considered in the index. Sub-indices for each food group were calculated, standardized and weighted according to the recommended age- and sex-specific desirable food intakes for the respective food groups. The obtained sub-scores were transformed into an overall healthy nutrition score from zero to 100. A higher score indicates a healthier dietary behaviour [19].

‘*Fruit intake*’, ‘*vegetable intake*’ and ‘*energy-dense food intake*’ were defined by using the HuSKY sub-indices for the food groups ‘fruit’ (fresh fruits), ‘vegetable’ (raw, cooked, frozen and canned vegetables and salad) and ‘sweets/fatty snacks/soft drinks’ (sugared soft and energy drinks, sweets, chocolate, cake, sweet pastries, ice cream, salty snacks, potato chips, crackers, fried potatoes, French fries, grilled or curried sausages, hamburger, kebab), respectively. The fruit and vegetable indices are constructed as the ratios of overall fruit/vegetable intake consumed in grams per day (FFQ information) divided by the age- and sex-specific recommended amounts (boys: 11–13, 250 g; 13- < 14, 300 g; 14- < 18, 350 g; girls: 11–13, 250 g; 13- < 14, 260 g; 14- < 18, 300 g). The energy-dense food index is constructed as the cumulated number of portions/standard units of ‘sweets, fatty snacks and soft drinks’ consumed per day.

The KiGGS – FFQ was validated against the computerized diet history interview ‘DISHES’; an overall mean correlation coefficient of 0.53 was reported [14]. The KiGGS – FFQ provides only rough information on dietary intake and the information was therefore used to rank individuals instead of using the continuous outcomes for analysis. Using a standardized procedure, quintiles were calculated for each outcome index, stratified by sex. The upper limits of the 3^rd^ quintile was used for the health favourable food indices (HuSKY, ‘fruit’, ‘vegetable’) to define intake as ‘high’ and the lower limit of the 3^rd^ quintile was used for the health unfavourable ‘energy-dense food’ index to define intake as ‘low’. The following cut-points were used, for *HuSKY:* 55.1 score for boys and 57.6 for girls; for *‘fruit intake’*: 0.50 ratio for boys and 0.60 for girls; for *‘vegetable intake’*: 0.41 ratio for boys and 0.49 for girls; and for *‘energy-dense food intake’*: 4.68 portions/standard unit of drinks for boys and 3.25 for girls. Boys and girls who were classified as having ‘high’ or ‘low’ intake for the respective outcomes were merged into unisex binary variables.

### Physical activity

*‘Leisure time physical activity’* was assessed with the following questions: ‘In your leisure time, how often are you physically active in such a way that you really start sweating or get out of breath (e.g. exercising, bicycling etc.)?’Possible answers were: ‘nearly every day’, ‘3–5 times a week’, ‘about 1–2 times a week’, ‘about 1–2 times a month’ or ‘never’. The subsequent question was: ‘About how many hours is that approximately per week? __ __’.

*‘Media use’* was assessed with the question: ‘How much time do you spend on average per day doing the following? (1) Television/video, (2) video games, (3) computer/internet, (4) listening to music, (5) using cell phone. Answers were, not at all, about 30 min, about 1–2 h, about 3–4 h, more than 4 h’. A media use index was calculated by cumulating the amount of time spent on a daily basis with the respective activities.

*‘Total energy expenditure’* in 24 h was calculated from information on ‘leisure time physical activity’, ‘media use’ and ‘sleeping time’. Metabolic equivalent values (MET) were assigned to the activity categories, 0.9 MET for sleeping time, 1.3 MET for media use, 8 MET for leisure time physical activity [21]. It was assumed that the remainder of the 24 h period was spent on average with ‘light activities’ for which a MET value of 2.5 per hour was assigned [21]. The respective activity scores were summed up into a summary score of ‘total energy expenditure’ in MET hours per 24 h.

Tertiles were calculated for all physical activity indices stratified by sex, and the population was divided into three equal groups using the upper limits of the first and second tertile. The following cut-points were used. *Leisure time activity duration*: 4 and 8 h per week for boys, 2 and 5 for girls. *Media use duration:* 3.5 and 6.5 h per day for both boys and girls. *Total energy expenditure:* 43.0 and 47.3 METs per 24 h for boys, 41.3 and 45.0 for girls.

*‘Familial leisure activity’* was assessed by asking respondents to [1] ‘disagree’, (2) ‘rather disagree’, (3) ‘rather agree’ or (4) ‘agree’ with the statement, ‘As a family in the evening and at weekends we rather stay at home than doing leisure activities together’. The categories 1 and 2 were used to define familial leisure activity level as ‘high’ and the categories 3 and 4 as ‘low’. The question is a sub-item of the ‘Familienklimaskalen’ (FKS) [22], which is a translated and slightly adapted German version of the family environment scales (FES) [23].

### Weight status related variables

*‘BMI-for-age’* was calculated for boys and girls separately applying the ‘BMI-for-age’ reference z-scores of the World Health Organization (WHO) [24] using the following cut-points: ‘below -2 Z’, ‘-2 to -1 Z’, ‘-1 to +1 Z’, ‘+1 to +2 Z’, and ‘above +2 Z’ [25].

‘*Perceived weight status’* was assessed by asking the parent: ‘Do you think that your child is: (1) by far too thin; (2) too thin; (3) normal; (4) too fat; (5) by far too fat?’

### Statistical analysis

The statistical analyses were performed with STATA SE 12.1. Survey design procedures were used to adjust for the cluster design effect. Potential influencing factors for the investigated associations were identified and logistic regression analyses were performed to investigate their statistical significance: *Model 1*: outcome and exposure variable; *Model 2*: Model 1 + influencing factor; and *Model 3*: Model 2 + interaction term of exposure*influencing factor. If the influencing factor was associated with the exposure and independently associated with the outcome in Model 2, the influencing factor was considered in the multivariate analyses. If the interaction term was significant in Model 3, effect modification was assumed and sub group analysis was performed. Associations between PSEP and nutrition outcomes were analysed using multiple logistic regression models, conducting a separate analysis for each association between the exposures (education, occupation and income) and the outcome variables (fruit intake, vegetable intake, energy-dense food intake and HuSKY); the results were stratified by sex. The age and region adjusted associations in the basic models were subsequently adjusted for identified influencing factors in the final models. When adjusting for covariates we used the age strata 11–13, 14–15 and 16–17. We used the regional strata, ‘East’ and ‘West’ Germany, tertiles of the leisure time physical activity duration (in hours per week) as well as media use (in hours per day) and the total energy expenditure (in MET/24 h) in addition to the strata of ‘familial leisure activity’ ‘high’ and ‘low’; the ‘BMI-for-age’ strata, ‘below -2 Z’, ‘-2 to -1 Z’, ‘-1 to +1 Z’, ‘+1 to +2 Z’, ‘above +2 Z’ and the categories of ‘perceived weight status’ ‘(by far) too thin’, ‘normal’, ‘(by far) too fat’. Missing values of the covariates were included in the statistical analyses by generating a separate category for missing values. Finally mediation analysis was performed to investigate the role of covariates on the investigated associations between PSEP and dietary behaviour among adolescents using the Baron and Kenny criteria for mediation: the covariate is associated with the exposure, it is independently associated with the outcome and transmits the association between the exposure and the outcome (the association is smaller in absolute terms when controlling for the potential mediator) [26, 27]. The percentage of change in the ORs is calculated as ((model a–model b)/(model a-1)), where model a is the basic model and model b is the model including the hypothesized mediator variable. Parental education was used as PSEP exposure in the mediation analyses, since education is less likely to change after the end of the educational carrier and the item response rate was higher compared to those of occupation and income.

## Results

### Participants

Table 1 shows the distribution of the study sample according to selected covariates; all covariates, with the exception of age, were associated with parental education.

**Table 1:** Proportions of parental education according to covariates, adolescents aged 11–17 years, KiGGS 2003–2006

	Study Sample		Primary parental education	Secondary parental education	Tertiary parental education	
	n	%	% 95 % CI	% 95 % CI	% 95 % CI	Pearson’s chi^2^-test^a^
Total	6359		22	51	27	
Covariates						
Age group						
11–13	2892	45	22 (20–25)	51 (49–54)	26 (24–29)	
14–15	1834	29	22 (20–26)	52 (49–54)	26 (24–28)	
16–17	1633	26	21 (19–24)	50 (47–53)	29 (26–31)	*P* = 0.414
Region in Germany						
Former East	2160	34	5 (4–6)	64 (61–67)	31 (28–34)	
Former West	4199	66	26 (24–28)	48 (46–50)	26 (24–28)	*P* = 0.000
Leisure time physical activity						
Low	2384	37	22 (20–25)	50 (48–53)	27 (25–30)	
Middle	1849	29	20 (18–23)	50 (48–53)	30 (27–32)	
High	1794	28	22 (20–25)	53 (50–56)	25 (22–28)	
Missing	332	5	29 (23–35)	52 (46–58)	19 (15–25)	*P* = 0.003
Media use						
Low	2013	32	17 (15–90)	49 (46–52)	34 (31–37)	
Middle	2176	34	22 (20–25)	50 (48–53)	28 (25–31)	
High	1880	30	26 (23–29)	55 (52–57)	20 (18–22)	
Missing	290	5	29 (23–36)	50 (49–53)	21 (17–27)	*P* = 0.000
Total energy expenditure						
Low	1924	30	24 (21–27)	52 (50–55)	23 (21–26)	
Middle	1950	31	20 (18–23)	51 (48–54)	28 (26–31)	
High	1945	31	20 (17–22)	49 (47–52)	31 (28–34)	
Missing	540	8	28 (23–34)	51 (46–56)	21 (18–25)	*P* = 0.000
Familial leisure activity						
High	3354	53	18 (16–20)	51 (49–54)	32 (28–33)	
Low	2746	43	25 (22–27)	52 (49–54)	24 (22–26)	
Missing	259	4	41 (35–49)	40 (33–47)	19 (14–25)	*P* = 0.000
BMI-for-age						
Above +2Z	553	9	34 (29–39)	49 (44–53)	17 (14–21)	
+1 to +2 Z	1155	18	24 (21–27)	55 (51–58)	21 (19–24)	
–1 to +1Z	3941	62	20 (18–23)	51 (48–53)	29 (27–31)	
−2 to -1 Z	593	9	18 (15–22)	49 (45–54)	33 (28–37)	
Below -2Z	117	2	21 (13–32)	52 (41–62)	28 (19–37)	*P* = 0.000
Perceived weight status						
Fat	1537	24	25 (22–28)	53 (50–56)	22 (19–24)	
Normal	3654	57	21 (18–23)	50 (48–53)	29 (27–32)	
Thin	1084	17	21 (18–25)	51 (48–55)	28 (25–31)	
Missing	84	1	39 (29–51)	41 (31–52)	19 (11–31)	*P* = 0.000

^a^Test of trend for describing row differences for two-way tables with ordered column

### Multivariate analyses

The independent associations between the covariates and the nutrition outcome variables, adjusted for parental education, are shown in Table 2.

**Table 2:** Associations between nutrition indicators and covariates, adolescents aged 11–17 years, KiGGS 2003–2006, adjusted for parental education

	Study Sample	High fruit intake	High vegetable intake	Low intake of energy-dense food	High healthy nutrition score (HuSKY)
	n	OR 95 % CI	OR 95 % CI	OR 95 % CI	OR 95 % CI
Total	6359				
Covariates					
Age group					
11–13	2892	1.0	1.0	1.0	1.0
14–15	1834	0.8 (0.7–0.9)^a^	0.9 (0.8–1.1)	0.7 (0.6–0.8)^a^	0.8 (0.7–0.9)^a^
16–17	1633	0.7 (0.6–0.8)^a^	0.7 (0.6–0.7)	0.7 (0.6–0.8)^a^	0.6 (0.5–0.7)^a^
Region in Germany					
Former East	2160	1.0	1.0	1.0	1.0
Former West	4199	0.7 (0.6–0.8)^a^	1.3 (1.2–1.5)^a^	1.3 (1.1–1.5)^a^	0.8 (0.7–0.9)^a^
Leisure time physical activity					
Low	2384	1.0	1.0	1.0	1.0
Middle	1849	1.2 (1.0–1.3)^a^	1.3 (1.2–1.6)^a^	1.1 (1.0–1.3)	1.2 (1.1–1.4)^a^
High	1794	1.3 (1.2–1.5)^a^	1.3 (1.2–1.5)^a^	0.9 (0.8–1.0)	1.3 (1.1–1.5)^a^
Missing	332	1.2 (1.0–1.6)	1.4 (1.1–1.9)^a^	1.0 (1.0–1.3)	1.2 (0.9–1.6)
Media use					
Low	2013	1.0	1.0	1.0	1.0
Middle	2176	1.0 (0.9–1.1)	0.8 (0.7–1.0)^a^	0.7 (0.6–0.8)^a^	0.8 (0.7–0.9)^a^
High	1880	0.9 (0.8–1.1)	0.7 (0.6–0.8)^a^	0.4 (0.3–0.9)^a^	0.6 (0.5–0.7)^a^
Missing	290	1.3 (0.9–1.7)	1.0 (0.8–1.3)	0.7 (0.5–0.9)^a^	1.0 (0.7–1.3)
Total energy expenditure					
Low	1924	1.0	1.0	1.0	1.0
Middle	1950	1.3 (1.1–1.5)^a^	1.4 (1.2–1.6)^a^	1.7 (1.4–1.9)^a^	1.4 (1.2–1.6)^a^
High	1945	1.4 (1.2–1.6)^a^	1.6 (1.4–1.8)^a^	1.8 (1.5–2.1)^a^	1.8 (1.5–2.1)^a^
Missing	540	1.5 (1.2–1.9)^a^	1.5 (1.3–1.9)^a^	1.4 (1.1–1.8)^a^	1.5 (1.2–2.0)^a^
Familial leisure activity					
High	3354	1.0	1.0	1.0	1.0
Low	2746	1.0 (0.9–1.1)	0.9 (0.8–1.1)	0.8 (0.7–0.9)^a^	1.0 (0.9–1.1)
Missing	259	1.7 (1.3–2.2)^a^	0.8 (0.6–1.0)	0.6 (0.4–0.7)^a^	0.8 (0.6–1.0)^a^
BMI-for-age					
Above +2Z	553	1.0	1.0	1.0	1.0
+1 to +2 Z	1155	1.0 (0.8–1.3)	0.7 (0.6–0.9)^a^	0.7 (0.6–0.9)^a^	0.9 (0.7–1.1)
−1 to +1Z	3941	0.9 (0.7–1.1)	0.8 (0.7–1.0)	0.5 (0.4–0.7)^a^	0.7 (0.6–0.9)^a^
−2 to -1 Z	593	0.8 (0.6–1.1)	0.9 (0.7–1.2)	0.5 (0.3–0.6)^a^	0.8 (0.6–1.1)
Below -2Z	117	0.6 (0.4–1.1)	0.7 (0.4–1.1)	0.4 (0.2–0.7)^a^	0.8 (0.5–1.3)
Perceived weight status					
Fat	1537	1.0	1.0	1.0	1.0
Normal	3654	1.2 (1.0–1.4)^a^	1.1 (1.0–1.3)	1.4 (1.2–1.6)^a^	1.1 (0.9–1.3)
Thin	1084	1.3 (1.1–1.6)^a^	1.1 (0.9–1.3)	2.0 (1.6–2.4)^a^	1.3 (1.0–1.5)^a^
Missing	84	1.2 (0.7–1.8)	1.0 (0.6–1.7)	0.7 (0.4–1.4)	1.0 (0.6–1.6)

^a^Significant on a 95 % level of confidence

#### Fruit intake

Leisure time physical activity, TEE and perceived weight status were independently associated with fruit intake (Table 2). After adjustment (see Table 3), significant associations remained between parental education and fruit intake among boys and girls – with an OR of 1.3 (95 % CI 1.0–1.7; *p* = 0.031) for boys with secondary educated parents and 2.0 (1.5–2.7; *p* < 0.001) for boys with tertiary educated parents compared to boys with primary educated parents; the corresponding ORs among girls were 1.0 (0.8–1.4; *p* = 0.770) and 1.5 (1.0–2.1; *p* = 0.026).

**Table 3:** Odds ratios (ORs) of fruit and vegetable intake, by parental level of education, occupation and income among boys and girls aged 11–17 years, KiGGS 2003–2006

	High fruit intake				High vegetable intake			
	Basic Model^a^		Final Model^b^		Basic Model^a^		Final Model^c^	
	OR	95 % CI	OR	95 % CI	OR	95 % CI	OR	95 % CI
Boys (*n* = 3230)								
Parental education								
Primary	1.0		1.0		1.0		1.0	
Secondary	1.2	(0.9–1.5)	1.3	(1.0–1.7)^d^	0.9	(0.8–1.1)	0.9	(0.7–1.1)
Tertiary	1.6	(1.3–2.0)^d^	2.0	(1.5–2.7)^d^	1.5	(1.2–1.9)^d^	1.3	(1.0–1.7)^d^
Parental occupation								
Low	1.0		1.0		1.0		1.0	
Middle	0.9	(0.7–1.2)	0.8	(0.6–1.0)	1.1	(0.9–1.4)	1.1	(0.8–1.3)
High	1.0	(0.8–1.2)	0.8	(0.6–1.0)	1.4	(1.1–1.7)	1.2	(0.9–1.5)
Household income								
Low	1.0		1.0		1.0		1.0	
Middle	1.0	(0.8–1.2)	1.0	(0.8–1.2)	1.3	(1.1–1.5)	1.2	(1.0–1.4)
High	1.0	(0.8–1.2)	0.8	(0.7–1.1)	1.3	(1.0–1.6)	1.0	(0.8–1.3)
Girls (*n* = 3129)								
Parental education								
Primary	1.0		1.0		1.0		1.0	
Secondary	1.0	(0.8–1.4)	1.0	(0.8–1.4)	1.0	(0.8–1.3)	1.0	(0.8–1.3)
Tertiary	1.4	(1.1–1.9)^d^	1.5	(1.0–2.1)^d^	1.4	(1.1–1.8)^d^	1.3	(1.0–1.6)
Parental occupation								
Low	1.0		1.0		1.0		1.0	
Middle	1.0	(0.9–1.3)	1.0	(0.8–1.3)	1.2	(1.0–1.5)	1.1	(0.9–1.4)
High	1.2	(1.0–1.5)	1.0	(0.8–1.3)	1.3	(1.1–1.7)^d^	1.2	(0.9–1.5)
Household income								
Low	1.0		1.0		1.0		1.0	
Middle	1.0	(0.8–1.3)	1.0	(0.8–1.2)	1.1	(0.9–1.3)	1.0	(0.8–1.3)
High	1.1	(0.9–1.4)	1.0	(0.7–1.2)	1.2	(1.0–1.5)	1.0	(0.8–1.3)

^a^Model adjusted for age groups and region strata East–west Germany (separate models for education, occupation and income)

^b^Adjusted as the Basic Model and also for leisure time physical activity, total energy expenditure and perceived weight status (education, occupation and income in combined model)

^c^Adjusted as the Basic Model and also for leisure time physical activity, media use, total energy expenditure and BMI-for-age (education, occupation and income in combined model)

^d^Significant on a 95 % level of confidence

#### Vegetable intake

Leisure time physical activity, media use, TEE and BMI-for-age were independently associated with vegetable intake (Table 2). After adjustment (Table 3), a significant association remained between parental education and vegetable intake among boys – with an OR of 1.3 (1.0–1.7; *p* = 0.025) for boys with tertiary educated parents compared to boys with primary educated parents.

#### Energy-dense food intake

Media use, TEE, familial leisure activity, BMI-for-age and perceived weight status were independently associated with energy-dense food intake (Table 2). After adjustment (Table 4), significant associations remained between all PSEP indicators (education, occupation and income) and low intake of energy-dense food among boys as well as between parental education and parental occupation and low intake of energy-dense food among girls. The ORs for parental education were 1.3 (1.0–1.7; *p* = 0.044) for boys with secondary educated parents and 1.8 (1.4–2.3; *p* < 0.000) for boys with tertiary educated parents compared to boys with primary educated parents; the corresponding ORs among girls were 1.2 (0.9–1.5; *p* = 0.252) and 1.6 (1.2–2.2; *p* = 0.003).

**Table 4:** Odds ratios (ORs) of energy-dense food intake and Healthy Nutrition Score for Kids and Youth (HuSKY), by parental level of education, occupation and income among boys and girls aged 11–17 years, KiGGS 2003–2006

	Low intake of energy-dense food				High HuSKY			
	Basic Model^a^		Final Model^b^		Basic Model^a^		Final Model^c^	
	OR	95 % CI	OR	95 % CI	OR	95 % CI	OR	95 % CI
Boys (*n* = 3230)								
Parental education								
Primary	1.0		1.0		1.0		1.0	
Secondary	1.6	(1.2–2.0)^d^	1.3	(1.0–1.7)^d^	0.9	(0.7–1.1)	0.9	(0.7–1.2)
Tertiary	2.4	(1.9–3.0)^d^	1.8	(1.4–2.3)^d^	1.5	(1.2–1.9)^d^	1.6	(1.2–2.2)^d^
Parental occupation								
Low	1.0		1.0		1.0		1.0	
Middle	1.8	(1.5–2.2)^d^	1.4	(1.1–1.7)^d^	1.0	(0.8–1.2)	0.9	(0.7–1.1)
High	2.0	(1.6–2.4)^d^	1.3	(1.0–1.6)	1.1	(0.9–1.4)	0.9	(0.7–1.2)
Household income								
low	1.0		1.0		1.0		1.0	
middle	1.5	(1.2–1.8)^d^	1.3	(1.1–1.7)^d^	1.3	(1.1–1.5)^d^	1.2	(1.0–1.5)
high	1.9	(1.6–2.3)^d^	1.4	(1.1–1.7)^d^	1.1	(0.9–1.3)	0.9	(0.7–1.0)
Girls (*n* = 3129)								
Parental education								
Primary	1.0		1.0		1.0		1.0	
Secondary	1.3	(1.1–1.6)^d^	1.2	(0.9–1.5)	0.9	(0.8–1.2)	0.9	(0.7–1.1)
Tertiary	2.1	(1.6–2.7)^d^	1.6	(1.2–2.2)^d^	1.6	(1.3–2.1)^d^	1.5	(1.1–1.9)^d^
Parental occupation								
Low	1.0		1.0		1.0		1.0	
Middle	1.5	(1.2–1.9)^d^	1.3	(1.0–1.7)^d^	1.3	(1.0–1.6)	1.3	(1.0–1.6)
High	1.9	(1.5–2.3)^d^	1.3	(1.0–1.8)^d^	1.5	(1.2–1.8)^d^	1.2	(0.9–1.7)
Household income								
Low	1.0		1.0		1.0		1.0	
Middle	1.2	(1.0–1.4)	1.0	(0.8–1.2)	1.0	(0.8–1.3)	0.9	(0.7–1.2)
High	1.6	(1.3–1.9)^d^	1.1	(0.9–1.5)	1.1	(0.9–1.4)	0.9	(0.7–1.1)

^a^Model adjusted for age groups and region strata East–west Germany (separate models for education, occupation and income)

^b^Adjusted as the Basic Model and also for media use, total energy expenditure, familial leisure activity, BMI-for-age and perceived weight status (education, occupation and income in combined model)

^c^Adjusted as the Basic Model and also for leisure time physical activity, media use, total energy expenditure, BMI-for-age and perceived weight status (education, occupation and income in combined model)

^d^Significant on a 95 % level of confidence

#### HuSKY (Healthy Nutrition Score for Kids and Youth)

Leisure time physical activity, media use, TEE, BMI-for-age and perceived weight status were independently associated with the HuSKY (Table 2). After adjustment (Table 4), significant associations remained between parental education and the HuSKY among boys and girls – with an OR of 1.6 (1.2–2.2; *p* = 0.001) for boys with tertiary educated parents compared to boys with primary educated parents; the corresponding OR among girls was 1.5 (1.1–1.9; *p* = 0.011).

### Subgroup and mediation analyses

Perceived weight status was an effect modifier for the association between parental education and energy-dense food intake among girls (interaction term *p*-value 0.039, data not shown). The association was only significant in the stratum of girls perceived by their parents to be normal weight (Additional file 1: Table S1). TEE was an effect modifier for the association between parental education and the HuSKY among girls (interaction term *p*-value 0.012, data not shown). The association was only significant in the stratum of girls with high TEE (Additional file 1: Table S2).

Media use and familial leisure activity fulfilled the criteria of partial mediation for the association between parental education and energy-dense food intake. In the age and region adjusted model of the association between parental education and energy-dense food intake (Additional file 1: Table S3), media use explained 16 % of the association among boys and 24 % among girls when comparing adolescents with tertiary and primary parental educational backgrounds; the corresponding percentages were 13 % among boys and girls when comparing adolescents with tertiary and secondary parental educational backgrounds. In the age and region adjusted model of the association between parental education and energy-dense food intake familial leisure activity explained 6 % of the association among boys and 10 % among girls when comparing adolescents with tertiary and primary parental educational backgrounds (Additional file 1: Table S3); the corresponding percentages were 7 % among boys and 16 % among girls when comparing adolescents with tertiary and secondary parental educational backgrounds. Media use and TEE fulfilled the criteria of partial mediation for the association between parental education and the HuSKY. In the age and region adjusted model of the association between parental education and HuSKY, media use explained 11 % of the association among boys and 21 % among girls when comparing adolescents with tertiary and primary parental educational backgrounds (Additional file 1: Table S4). In the age and region adjusted model of the association between parental education and HuSKY (Additional file 1: Table S4), TEE explained 9 % of the association among boys and 20 % among girls when comparing adolescents with tertiary and primary parental educational backgrounds.

When weight related variables (BMI-for-age, perceived weight status) were included in the models, the general trend was that the association between parental education and nutrition outcomes became slightly stronger (data not shown).

## Discussion

In this large, population-representative sample of German adolescents it is observed that, after full adjustment, boys and girls with high-SEP parents consume more fruits, more vegetables (only boys), less energy-dense foods, and have a more healthy overall dietary behaviour than those with low-SEP parents. These observations are in line with the findings of other studies [8, 28–30]. The associations become weaker when controlling for physical activity variables and slightly stronger when controlling for body weight related variables. Parental education shows stronger independent associations with the nutrition outcomes than parental occupation and household income; this also confirms the findings of previous studies [31–33].

The levels of media use and familial leisure activity explained, in part, the variance of the observed associations between PSEP and high energy-dense food intake among adolescents. Media use mediated about a quarter and familial leisure activity a sixth of the total association when comparing girls with tertiary and primary educated parents. Also Scully et al. investigated physical activity and dietary behaviour among adolescents across SEP groups and found very similar patterns to those we observed [34]. Adolescents with low SEP background more frequently consumed fast food and high-energy drinks and spent more time on the internet, playing video games and watching TV. In addition, it was observed that adolescents who watched TV for more than 2 h per day consumed less fruits and more fast food, snacks and high-energy drinks, when controlling for SEP, physical activity and other variables [34]. Also other studies suggest that sedentary screen-time activity coincides with consumption of energy-dense snacks [35–37] and that reducing media use may automatically lead to a reduction of energy-dense food consumption among adolescents [38]. However, while technology developments have often led to reduction of physical activity in recent years (cars, television, video games), some developments like fitness tools and training or dietary advice apps may help to improve physical activity and reduce obesity.

**Fig. 1:**
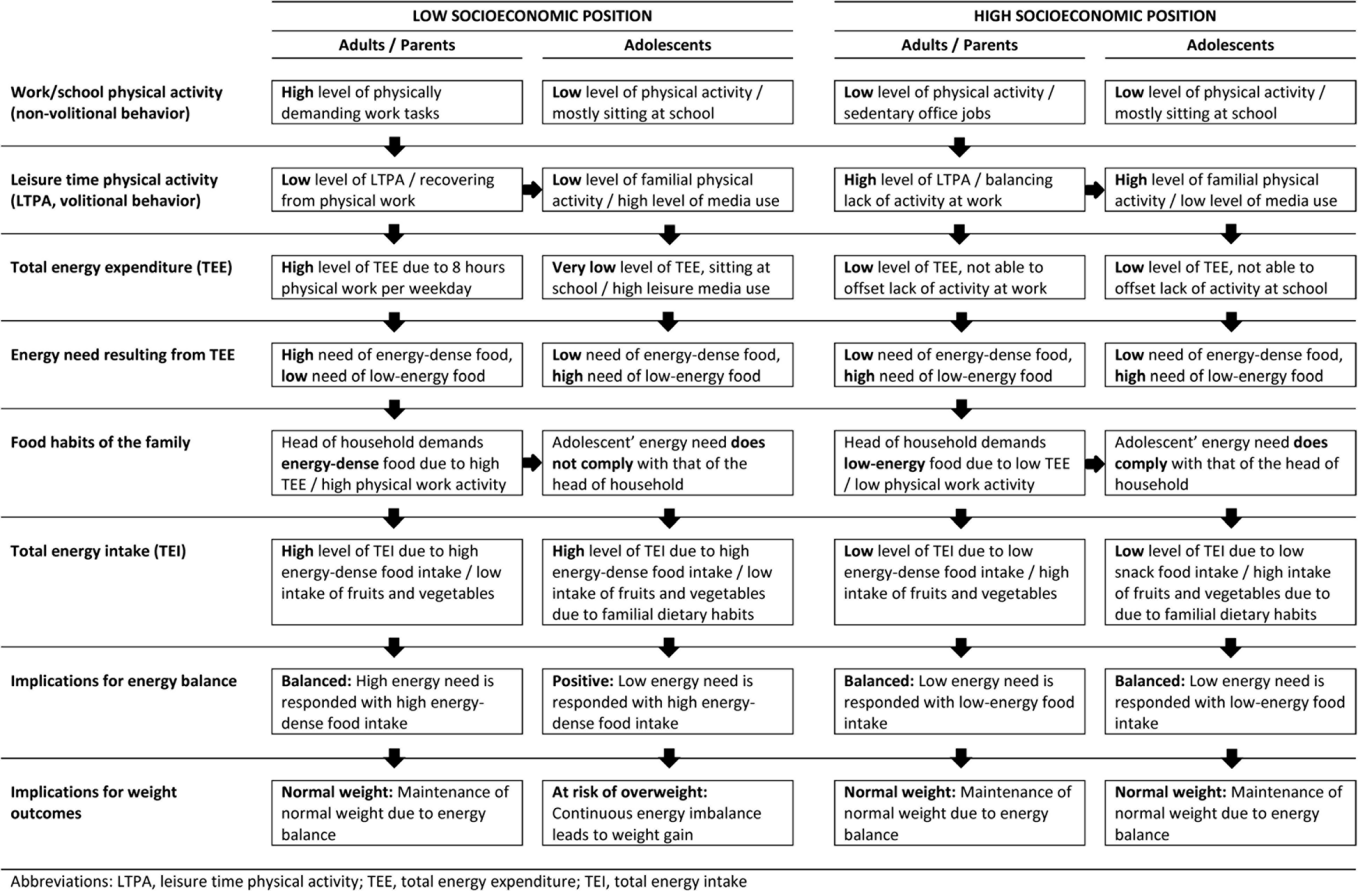
Potential pathways of adolescents dietary behaviour according to parental socioeconomic position

These findings are different from those observed among German adults for whom the association between level of education and energy-dense food intake was partly explained by more vigorous work activity (higher TEE) among the lower compared to the higher educated [6], whereas, among adolescents, the same association is partly explained by a higher level of media use and a lower level of familial leisure activity (lower TEE). Potential pathways showing how SEP may influence physical activity and diet related behaviours among adolescents and their parents are presented in Fig. 1. Parents with low education often have more physically-demanding jobs and, in order to recover from physical work, they may stay at home in their leisure time more often which may also imply lower familial leisure time activity compared to parents with high education, who mostly sit at work (office jobs) [4]. Partly because of this, their children may have higher media consumption and lower TEE than adolescents with high education backgrounds [5]. In high-SEP families physical activity patterns of the parents and their children may be more similar, dominated by sitting at work or school. Assuming that familial dietary habits are mainly controlled by the parents, and that physical workers need more high caloric food to compensate for their high work-related energy expenditure [6], their children may also consume more high caloric food compared with the adolescent offspring of sedentary workers. Hence, the dietary intake among adolescents in physical worker families (mainly low SEP families) may correspond less to their own energy requirements. As a result, adolescents with low-SEP parents may be at higher risk to develop overweight or obesity compared to adolescents with high-SEP parents.

In line with this hypothesis, studies have shown that energy-dense food availability is greater in low-PSEP families [39], that the food habits of children are related to those of their parents [8, 40] and that the children of farmers and blue collar workers consume more energy-dense foods than the children of white collar workers [32, 41, 42]. In addition, Martens et al. have shown that the consumption of high-fat snacks among Danish adolescents was negatively associated with food availability and accessibility and positively associated with parental behaviour [43]. In general when children get older the parental control on their dietary behaviour will becomes weaker, since they consume more foods away from home. A study of Lilico et al. in a population-based sample of Canadian youth shows that the prevalence of eating meals with an adult family member is lower and the prevalence of eating away from home in a fast-food restaurant is higher among 9–12 grade pupils compared to 5-8 grade pupils [44].

Although media use and familial leisure time activity levels partly explained the association between indicators of PSEP and energy-dense foods among adolescents, the associations remained significant after adjustment. Other studies suggest that, apart from food accessibility, food prices, the food environment, knowledge and culture may shape SEP differences in diet quality [30].

For instance, the perceived local food environment, in the manner that distance to get to shops and restaurants to buy healthy or unhealthy foods, as well as more or less healthy food offerings within shop, may not be the same for families living in deprived versus wealthy neighbourhoods [45]. Also perceived high cost of healthy food by low-SEP adults can have an influence on their children’s dietary behaviour [46]. Studies reviewed by Rao et al. show however, that healthier foods are not always more expensive; for example healthier options per serving for dairy foods were less expensive than the unhealthier options [47].

In addition, several other factors determine the dietary behaviour of adolescents [8]. These factors, which may have also influenced the results and for which we were not able to control, include personal, family-related, peer group-related and school-related factors.

Obesity can be a consequence of an unhealthy diet, but also a motivation to adopt a healthier dietary behaviour. Therefore and because our study is cross-sectional, we considered it as a potential influencing factor in our study. However, the statistical analysis revealed that the body weight related variables did not act as mediators according to the Baron and Kenny criteria of mediation [27]. An exception was the perceived weight status which was an effect modifier for the association between PSEP and overall healthy food intake among girls. A significant association was only observed among girls perceived by their parents to be ‘normal weight’, but not among girls perceived to be ‘too fat’. Low-SEP parents may perceive their girls as normal weight, although objectively they are overweight more often than what high-SEP parents perceive [48, 49]. The parental perception of unhealthy body weight to their female offspring seems to have an influence on the dietary behaviour of their children [50]. A starting point for health interventions could be to increase the parental awareness of the problematic weight status of their children, particularly in low-SEP families.

In general, we observed that among girls the influence of physical activity and weight status related covariates on the associations between PSEP and dietary behaviour is stronger than among boys. This finding is in line with other studies in this field, which observed for instance stronger associations between calories chosen by the mother for their children and mother’s education, mother’s weight status, mother’s obesity genetic causal beliefs, mother’s perceived importance of the amount of fast food intake in causing childhood obesity and mother’s restriction of her child’s food intake among girls than among boys [51]. Several potential reasons may explain the observed gender differences in our study. Firstly, the validation of the KiGGS FFQ showed that the self reports of girls showed a higher validity for some food groups compared to those of boys [14]. This may result in a higher type 2 bias among boys and may distort possible interactions in the boys. Secondly, the self reports of the parents on perceived weight status seem to be less valid for the boys than for the girls. While parental perception of the child’s weight status conforms well to the BMI-for-age among girls: ‘Too fat’: 26.05 %; ‘BMI-fo0r-age ≥ +1Z’: 25.02 %, the corresponding percentages among boys were less in line: 22.35 % and 28.64 %. It seems that parents more strongly underestimate obesity among boys than among girls [49], which may lead to a stronger type 2 bias among the boys. Thirdly, the explanatory power of a mediator is stronger where the association between the exposure (PSEP) and the mediator (physical activity and weight related variables) is stronger. In our previous study [5], we demonstrated much stronger associations between PSEP and physical activity outcome indicators (media use, leisure time physical activity, TEE) for girls than for boys. This might be another explanation why the mediation effects of physical activity indicators were stronger among girls compared to boys.

### Limitations

Food frequency assessment based on self-reporting has several limitations [52]: Healthy food intake may be over reported and unhealthy food intake may be under reported due to social desirability bias. The relative validation of the KiGGS – FFQ, with intake assessed using more detailed dietary histories, revealed that the correlation between both methods differed according to sex, age, body weight and socio-economic status for specific food groups [14]. The adolescents with normal weight, for instance, had a higher correlation coefficient (0.54 [0.49–0.58]) for ‘sweets’ than overweight adolescents (0.36 [0.23–0.47]). We cannot fully exclude the possibility of differential misclassification bias in this study. Physical activity assessment based on self-reporting has limitations [53], which can cause type 2 bias, where a true association is not detected (false negative). This may be a reason why the inclusion of physical activity variables in the final models did not change the ORs significantly. We selected the covariates based on prior knowledge with references from the literature and performed a stepwise selection procedure with univariate pre-screening of potential covariates. Although these strategies are the most commonly used methods, they have been criticized for their limitations [54]. We performed an additional analyses were we included all covariates shown in Table 2 into the final models; the ORs only slightly changed on the level of the second decimal place compared to the stepwise procedure.

The Baron and Kenny traditional approach has been criticized because the influences of additional variables, which could act as mediator-outcome confounders, are not considered [55]. We performed another mediation analysis to check whether a different approach will produce other results and considered the influence of additional variables during the analysis. For all mediation analyses shown in the Additional file 1: Table S3 and Additional file 1: Table S4 the overall findings remained essentially unchanged. An exception was the association between parental education and HuSKY (Additional file 1: Table S4) where media use was no longer significantly associated with the HuSKY among boys and girls when TEE was considered.

Finally, the response analysis has shown that respondents differ from the non-respondents according to the parental education level [5], which possibly further compromises the validity of the results.

## Conclusions

In Germany, adolescents with low SEP parents showed a lower compliance with the national nutrition recommendations on fruit intake, vegetable intake and energy-dense food intake and also a lower compliance with overall healthy nutrition compared to adolescents with high SEP parents. Higher levels of media use and lower levels of familial leisure time activity among adolescents with low PSEP may partly explain why they consume more energy-dense food. The higher energy-dense food intake of adolescents with low SEP parents seems not to be linked to higher levels of TEE. Rather it may depend on family-related factors and a less supportive environment for having an adequate energy diet. Preferably school-based interventions are needed to promote a low intake of unhealthy energy-dense food among German adolescents. In order to prevent health inequalities in future, a special effort is needed to reach adolescents from low-SEP families. A combined strategy of promoting a low energy diet and physical activity at the same time could be an effective strategy to prevent the development of obesity among adolescents coming from low-SEP families.

## Additional file

Additional file 1:
**Contains the results of the association between parental education and energy-dense food intake stratified by perceived weight status** **(Table S1)** **, of the association between parental education and HuSKY stratified by total energy expenditure** **(Table S2)** **, and the mediation analyses of the associations between parental education and** **energy-dense food intake adjusted for media use and familial leisure activity** **(Table S3)** **and of the association between parental education and HuSKY adjusted for media use and total energy expenditure** **(Table S4)** **, as well as the illustration of the assessment method of level of education (Fig. S1).** Adobe Acrobat Reader is required to access this file.

## Abbreviations

BMI: Body mass index
CASMIN: Comparative Analysis of Social Mobility in Industrial Nations
FES: Family Environment Scales
FFQ: Food frequency questionnaire
FKS: Familienklimaskalen
HuSKY: Healthy Nutrition Score for Children and Youth
KiGGS: German Health Interview and Examination Survey for Children and Adolescents (Kinder- und Jugendgesundheitssurvey)
MET: Metabolic equivalent
OECD: The Organisation for Economic Co-operation and Development
OMD: Optimized mixed diet
PSEP: Parental socioeconomic position
SEP: Socioeconomic position
TEE: Total energy expenditure
WHO: World Health Organization

## References

[1] Word Health Organization (2003). Diet, nutrition and the prevention of chronic diseases. report of a joint WHO/FAO expert consultation. World health organization and agricultural organization of the United Nations.

[2] Vlismas K, Stavrinos V, Panagiotakos DB (2009). Socio-economic status, dietary habits and health-related outcomes in various parts of the world: a review. Cent Eur J Public Health.

[3] Schutz Y, Caballero B (2005). Energy balance. Encyclopedia of human nutrition.

[4] Finger JD, Tylleskär T, Lampert T, Mensink GBM (2012). Physical activity patterns and socioeconomic position: the German National Health Interview and Examination Survey 1998 (GNHIES98). BMC Public Health.

[5] Finger JD, Mensink GBM, Lampert T, Tylleskär T (2014). Physical activity, aerobic fitness and parental socioeconomic position among adolescents: the German health interview and examination for children and adolescents 2003-2006 (KiGGS). Int J Behav Nutr Phys Act.

[6] Finger JD, Tylleskär T, Lampert T, Mensink GBM (2013). Dietary behaviour and socioeconomic position: the role of physical activity patterns. PLoS One.

[7] Hanson MD, Chen E (2007). Socioeconomic status and health behaviors in adolescence: a review of the literature. J Behav Med.

[8] Rasmussen M, Krølner R, Klepp KI, Lytle L, Brug J, Bere E (2006). Determinants of fruit and vegetable consumption among children and adolescents: a review of the literature. Part I: quantitative studies. Int J Behav Nutr Phys Act..

[9] Kleiser C, Schaffrath-Rosario A, Mensink GBM, Prinz-Langenohl R, Kurth BM (2009). Potential determinants of obesity among children and adolescents in Germany: results from the cross-sectional KiGGS Study. BMC Public Health.

[10] Galobardes B, Shaw M, Lawlor DA, Lynch JW, Davey Smith G (2006). Indicators of socioeconomic position (part 1). J Epidemiol Community Health.

[11] Kurth BM, Schaffrath Rosario A (2010). [Overweight and obesity in children and adolescents in Germany]. Bundesgesundheitsblatt Gesundheitsforschung Gesundheitsschutz.

[12] Kamtsiuris P, Lange M, Schaffrath Rosario A (2007). [The German health interview and examination survey for children and adolescents (KiGGS): sample design, response and nonresponse analysis]. Bundesgesundheitsblatt Gesundheitsforschung Gesundheitsschutz.

[13] Kurth BM, Kamtsiuris P, Hölling H, Schlaud M, Dölle R, Ellert U (2008). The challenge of comprehensively mapping children’s health in a nation-wide health survey: design of the German KiGGS-Study. BMC Public Health.

[14] Truthmann J, Mensink GBM, Richter A (2011). Relative validation of the KiGGS food frequency questionnaire among adolescents in Germany. Nutr J.

[15] Schroedter JH, Lechert Y, Lüttinger P (2006). Die Umsetzung der Bildungsklassifikation CASMIN für die Volkszählung 1970, die Mikrozensus-Zusatzerhebung 1971 und die Mikrozensen 1976–2004 [Transformation of the CASMIN education classification for the census 1970, the micro-census supplement 1971 and the micro-censuses 1976–2004]. ZUMA Methodenbericht..

[16] Müller W, Lüttinger P, König W, Karle W (1989). Class and education in industrial nations. Int J Sociol.

[17] Hoffmeyer-Zlotnik JHP, Geis AJ (2003). Berufsklassifikation und messung des beruflichen status/prestige [occupational classification and measurement of occupational status/prestige]. ZUMA-Nachrichten.

[18] OECD. OECD: Project on Income Distribution and Poverty. What are equivalent scales? Available from: http://www.oecd.org/eco/growth/OECD-Note-EquivalenceScales.pdf (accessed: 3 December 2011). 2011.

[19] Kleiser C, Mensink G, Scheidt-Nave C, Kurth BM (2009). HuSKY: a healthy nutrition score based on food intake of children and adolescents in Germany. Br J Nutr.

[20] Kersting M, Sichert-Hellert W, Vereecken CA, Diehl J, Beghin L, De Henauw S (2008). Food and nutrient intake, nutritional knowledge and diet-related attitudes in European adolescents. Int J Obes (Lond).

[21] Ridley K, Ainsworth B, Olds T (2008). Development of a compendium of energy expenditures for youth. Int J Behav Nutr Phys Act.

[22] Schneewind K, Cierpka M (1988). Die Familienklimaskalen (FKS) [The family environment scales]. Familiendiagnostik.

[23] Moos RH, Moos BS (1994). Family environment scale manual.

[24] World Health Organization: WHO Reference 2007. Growth reference 5–19 years. Available from: http://www.who.int/growthref/en/ (accessed: 8 January 2013). 2007.

[25] Onis M, Onyango AW, Borghi E, Siyam A, Nishida C, Siekmann J (2007). Development of a WHO growth reference for school-aged children and adolescents. Bull World Health Organ.

[26] MacKinnon DP, Fairchild AJ, Fritz MS (2007). Mediation analysis. Annu Rev Clin Psychol.

[27] Baron RM, Kenny DA (1986). The moderator-mediator variable distinction in social psychological research: conceptual, strategic, and statistical considerations. J Pers Soc Psychol.

[28] Van Der Horst K, Oenema A, Ferreira I, Wendel-Vos W, Giskes K, Van Lenthe F (2007). A systematic review of environmental correlates of obesity-related dietary behaviors in youth. Health Educ Res.

[29] Cameron AJ, Ball K, Pearson N, Lioret S, Crawford DA, Campbell K (2012). Socioeconomic variation in diet and activity-related behaviours of Australian children and adolescents aged 2–16 years. Pediatr Obes.

[30] Darmon N, Drewnowski A (2008). Does social class predict diet quality?. Am J Clin Nutr.

[31] Goodwin DK, Knol LK, Eddy JM, Fitzhugh EC, Kendrick O, Donohue RE (2006). Sociodemographic correlates of overall quality of dietary intake of US adolescents. Nutr Res.

[32] Laitinen S, Räsänen L, Viikari J, Åkerblom HK (1995). Diet of Finnish children in relation to the family’s socio-economic status. Scand J Public Health.

[33] Nilsen SM, Krokstad S, Holmen TL, Westin S (2010). Adolescents’ health-related dietary patterns by parental socio-economic position, The Nord-Trøndelag Health Study (HUNT). Eur J Publ Health.

[34] Scully M, Dixon H, White V, Beckmann K (2007). Dietary, physical activity and sedentary behaviour among Australian secondary students in 2005. Health Promot Int.

[35] Pearson N, Biddle SJH (2011). Sedentary behavior and dietary intake in children, adolescents, and adults: a systematic review. Am J Prev Med.

[36] Ranjit N, Evans MH, Byrd-Williams C, Evans AE, Hoelscher DM (2010). Dietary and activity correlates of sugar-sweetened beverage consumption among adolescents. Pediatrics.

[37] Utter J, Neumark-Sztainer D, Jeffery R, Story M (2003). Couch potatoes or French fries: are sedentary behaviors associated with body mass index, physical activity, and dietary behaviors among adolescents?. J Am Diet Assoc.

[38] Robinson TN (1999). Reducing children’s television viewing to prevent obesity: a randomized controlled trial. JAMA.

[39] Bjelland M, Lien N, Grydeland M, Bergh IH, Anderssen SA, Ommundsen Y (2011). Intakes and perceived home availability of sugar-sweetened beverages, fruit and vegetables as reported by mothers, fathers and adolescents in the HEIA (HEalth In Adolescents) study. Public Health Nutr.

[40] Wang Y, Beydoun MA, Li J, Liu Y, Moreno LA (2011). Do children and their parents eat a similar diet? resemblance in child and parental dietary intake: systematic review and meta-analysis. J Epidemiol Community Health.

[41] Haapalahti M, Mykkanen H, Tikkanen S, Kokkonen J (2003). Meal patterns and food use in 10 to 11 year-old Finnish children. Public Health Nutr.

[42] Prättälä R, Rahkonen O, Rimpelä M (1986). Consumption patterns of critical fat sources among adolescents in 1977–1985. Nutr Res.

[43] Martens M, Van Assema P, Brug J (2005). Why do adolescents eat what they eat? personal and social environmental predictors of fruit, snack and breakfast consumption among 12–14 year-old Dutch students. Public Health Nutrition-Wallingford.

[44] Lillico H, Hammond D, Manske S, Murnaghan D (2014). The prevalence of eating behaviors among Canadian youth using cross-sectional school-based surveys. BMC Public Health.

[45] Inglis V, Ball K, Crawford D (2008). Socioeconomic variations in women’s diets: what is the role of perceptions of the local food environment?. J Epidemiol Community Health.

[46] Williams L, Abbott G, Crawford D, Ball K (2012). Associations between mothers’ perceptions of the cost of fruit and vegetables and children’s diets: will children pay the price?. Eur J Clin Nutr.

[47] Rao M, Afshin A, Singh G, Mozaffarian D (2013). Do healthier foods and diet patterns cost more than less healthy options? a systematic review and meta-analysis. BMJ Open.

[48] Jain A, Sherman SN, Chamberlin LA, Carter Y, Powers SW, Whitaker RC (2001). Why don’t low-income mothers worry about their preschoolers being overweight?. Pediatrics.

[49] Lundahl A, Kidwell KM, Nelson TD (2014). Parental underestimates of child weight: a meta-analysis. Pediatrics.

[50] Rhee KE, De Lago CW, Arscott-Mills T, Mehta SD, Davis RK (2005). Factors associated with parental readiness to make changes for overweight children. Pediatrics.

[51] Bouhlal S, McBride CM, Ward DS, Persky S (2015). Drivers of overweight mothers’ food choice behaviors depend on child gender. Appetite.

[52] Brener ND, Billy JOG, Grady WR (2003). Assessment of factors affecting the validity of self-reported health-risk behavior among adolescents: evidence from the scientific literature. J Adolesc Health.

[53] Helmerhorst HJ, Brage S, Warren J, Besson H, Ekelund U (2012). A systematic review of reliability and objective criterion-related validity of physical activity questionnaires. Int J Behav Nutr Phys Act.

[54] Walter S, Tiemeier H (2009). Variable selection: current practice in epidemiological studies. Eur J Epidemiol.

[55] Richiardi L, Bellocco R, Zugna D (2013). Mediation analysis in epidemiology: methods, interpretation and bias. Int J Epidemiol.

