# Supplementary material for: Dietary behaviour and parental socioeconomic position among adolescents: the German Health Interview and Examination Survey for Children and Adolescents 2003–2006 (KiGGS)
Source: BMC Public Health. 2015 May 19;15:498. doi: 10.1186/s12889-015-1830-2 (PMC4492169; doi:10.1186/s12889-015-1830-2)
Supplement: Additional file 1: — Contains the results of the association between parental education and energy-dense food intake stratified by perceived weight status (Table S1) , of the association between parental education and HuSKY stratified by total energy expenditure (Table S2) , and the mediation analyses of the associations between parental education and energy-dense food intake adjusted for media use and familial leisure activity (Table S3) and of the association between parental education and HuSKY adjusted for media use and total energy expenditure (Table S4) , as well as the illustration of the assessment method of level of education (Fig. S1). Adobe Acrobat Reader is required to access this file. [file 12889_2015_1830_MOESM1_ESM.docx]

**Additional File**

**Tables**

| **Table 1. Odds ratios (OR) of the association between parental education and energy-dense food intake stratified by perceived weight status, girls aged 11-17** | | | |
| --- | --- | --- | --- |
|  | **No. in sample** |  | **Low intake of energy-dense food** |
|  |  |  | OR 95% CI |
|  |  |  |  |
| **Stratified analysis by perceived weight status ^a^** |  |  |  |
| **fat** | 815 |  |  |
| Primary education | 176 |  | 1.0 |
| Secondary education | 467 |  | 1.2 (0.8-1.8) |
| Tertiary education | 172 |  | 1.6 (1.0-2.5) |
|  |  |  |  |
| **normal** | 1896 |  |  |
| Primary education | 317 |  | 1.0 |
| Secondary education | 1021 |  | 1.4 (1.0-2.0)* |
| Tertiary education | 558 |  | 2.6 (1.8-3.6)* |
|  |  |  |  |
| **thin** | 382 |  |  |
| Primary education | 76 |  | 1.0 |
| Secondary education | 207 |  | 1.3 (0.6-2.8) |
| Tertiary education | 99 |  | 1.9 (0.8-4.7) |
|  |  |  |  |
| ^a^ Models adjusted for age groups and regional strata east vs. west Germany. * Significant on a 95% level of confidence. | | | |

| **Table 2. Odds ratios (OR) of the association between parental education and HuSKY stratified by total energy expenditure, girls aged 11-17** | | | |
| --- | --- | --- | --- |
|  | **No. in sample** |  | **High HuSKY** |
|  |  |  | OR 95% CI |
| **Stratified analysis by total energy expenditure ^a^** |  |  |  |
| **low** | 947 |  |  |
| Primary education | 197 |  | 1.0 |
| Secondary education | 548 |  | 1.2 (0.8-1.7) |
| Tertiary education | 202 |  | 1.2 (0.8-2.09) |
|  |  |  |  |
| **middle** | 961 |  |  |
| Primary education | 180 |  | 1.0 |
| Secondary education | 526 |  | 0.7 (0.5-1.1) |
| Tertiary education | 255 |  | 1.3 (0.8-2.1) |
|  |  |  |  |
| **high** | 955 |  |  |
| Primary education | 143 |  | 1.0 |
| Secondary education | 497 |  | 1.0 (0.7-1.5) |
| Tertiary education | 315 |  | 2.2 (1.5-3.4)* |
|  |  |  |  |
| ^a^ Models adjusted for age groups and regional strata east vs. west Germany. * Significant on a 95% level of confidence. | | | |

| **Table 3. Mediation analyses of the association between parental education and low intake of energy-dense food ^a^ adjusted for media use and familial leisure activity, boys and girls aged 11-17** | | | | | |
| --- | --- | --- | --- | --- | --- |
|  | **Basic Model; age + region + parental education** |  | **Model 1; Basic Model + media use** |  | **Model 2; Basic Model + familial leisure activity** |
|  | OR 95% CI P-value |  | OR 95% CI P-value |  | OR 95% CI P-value |
| **Boys** |  |  |  |  |  |
| **Parental education** |  |  |  |  |  |
| primary | 1.0 |  | 1.0 |  | 1.0 |
| secondary | 1.55 (1.22-1.97) 0.000 |  | 1.48 (1.16-1.90) 0.002 |  | 1.51 (1.19-1.91) 0.001 |
| tertiary | 2.41 (1.95-2.99) 0.000 |  | 2.18 (1.75-2.72) 0.000 |  | 2.32 (1.88-2.88) 0.000 |
|  |  |  |  |  |  |
| **Media use** |  |  |  |  |  |
| low |  |  | 1.0 |  |  |
| middle |  |  | 0.67 (0.55-0.81) 0.000 |  |  |
| High |  |  | 0.45 (0.36-0.57) 0.000 |  |  |
| missing |  |  | 0.67 (0.44-1.00) 0.053 |  |  |
|  |  |  |  |  |  |
| **Familial leisure activity** |  |  |  |  |  |
| high |  |  |  |  | 1.0 |
| low |  |  |  |  | 0.87 (0.74-1.03) 0.100 |
| missing |  |  |  |  | 0.64 (0.41-0.99) 0.043 |
|  |  |  |  |  |  |
| **Girls** |  |  |  |  |  |
| **Parental education** |  |  |  |  |  |
| primary | 1.0 |  | 1.0 |  | 1.0 |
| secondary | 1.31 (1.05-1.64) 0.017 |  | 1.27 (1.01-1.59) 0.038 |  | 1.26 (1.01-1.57) 0.037 |
| tertiary | 2.10 (1.65-2.67) 0.000 |  | 1.84 (1.44-2.35) 0.000 |  | 1.99 (1.56-2.54) 0.000 |
|  |  |  |  |  |  |
| **Media use** |  |  |  |  |  |
| low |  |  | 1.0 |  |  |
| middle |  |  | 0.72 (0.60-0.87) 0.001 |  |  |
| high |  |  | 0.38 (0.31-0.47) 0.000 |  |  |
| missing |  |  | 0.82 (0.54-1.23) 0.326 |  |  |
|  |  |  |  |  |  |
| **Familial leisure activity** |  |  |  |  |  |
| high |  |  |  |  | 1.0 |
| low |  |  |  |  | 0.82 (0.70-0.97) 0.018 |
| missing |  |  |  |  | 0.45 (0.29-0.69) 0.000 |
| ^a^ Energy-dense food intake is defined as ‘low’ using the lower limit of the 3^rd^ quintile as the cut-off point dividing the population in 40% versus 60%. | | | | | |

| **Table 4. Mediation analyses of the association between parental education and a high HuSKY ^a^ adjusted for media use and total energy expenditure, boys and girls aged 11-17** | | | | | |
| --- | --- | --- | --- | --- | --- |
|  | **Basic Model; age + region + parental education** |  | **Model 1; Basic Model + media use** |  | **Model 2; Basic Model + total energy expenditure** |
|  | OR 95% CI P-value |  | OR 95% CI P-value |  | OR 95% CI P-value |
| **Boys** |  |  |  |  |  |
| **Parental education** |  |  |  |  |  |
| primary | 1.0 |  | 1.0 |  | 1.0 |
| secondary | 0.89 (0.72-1.11) 0.294 |  | 0.87 (0.71-1.09) 0.222 |  | 0.88 (0.71-1.09) 0.237 |
| tertiary | 1.47 (1.15-1.88) 0.002 |  | 1.42 (1.11-1.81) 0.006 |  | 1.43 (1.12-1.82) 0.005 |
|  |  |  |  |  |  |
| **Media use** |  |  |  |  |  |
| low |  |  | 1.0 |  |  |
| middle |  |  | 0.84 (0.69-1.04) 0.103 |  |  |
| high |  |  | 0.77 (0.62-0.95) 0.017 |  |  |
| missing |  |  | 0.83 (0.55-1.26) 0.380 |  |  |
|  |  |  |  |  |  |
| **Total energy expenditure** |  |  |  |  |  |
| low |  |  |  |  | 1.0 |
| middle |  |  |  |  | 1.41 (1.14-1.73) 0.001 |
| high |  |  |  |  | 1.46 (1.18-1.80) 0.001 |
| missing |  |  |  |  | 1.19 (0.83-1.68) 0.340 |
|  |  |  |  |  |  |
| **Girls** |  |  |  |  |  |
| **Parental education** |  |  |  |  |  |
| primary | 1.0 |  | 1.0 |  | 1.0 |
| secondary | 0.94 (0.75-1.18) 0.589 |  | 0.92 (0.74-1.16) 0.484 |  | 0.91 (0.73-1.14) 0.408 |
| tertiary | 1.61 (1.26-2.06) 0.000 |  | 1.49 (1.16-1.91) 0.002 |  | 1.49 (1.17-1.91) 0.002 |
|  |  |  |  |  |  |
| **Media use** |  |  |  |  |  |
| low |  |  | 1.0 |  |  |
| middle |  |  | 0.75 (0.61-0.94) 0.011 |  |  |
| high |  |  | 0.57 (0.46-0.71) 0.000 |  |  |
| missing |  |  | 1.14 (0.75-1.74) 0.544 |  |  |
|  |  |  |  |  |  |
| **Total energy expenditure** |  |  |  |  |  |
| low |  |  |  |  | 1.0 |
| middle |  |  |  |  | 1.34 (1.08-1.65) 0.007 |
| high |  |  |  |  | 1.95 (1.59-2.39) 0.000 |
| missing |  |  |  |  | 1.77 (1.28-2.43) 0.001 |
| ^a^ Healthy Nutrition Score for Kids and Youth (HuSKY) is defined as ‘high’ using the upper limit of the 3^rd^ quintile as the cut-off point dividing the population in 40% versus 60%. | | | | | |

**Figures**


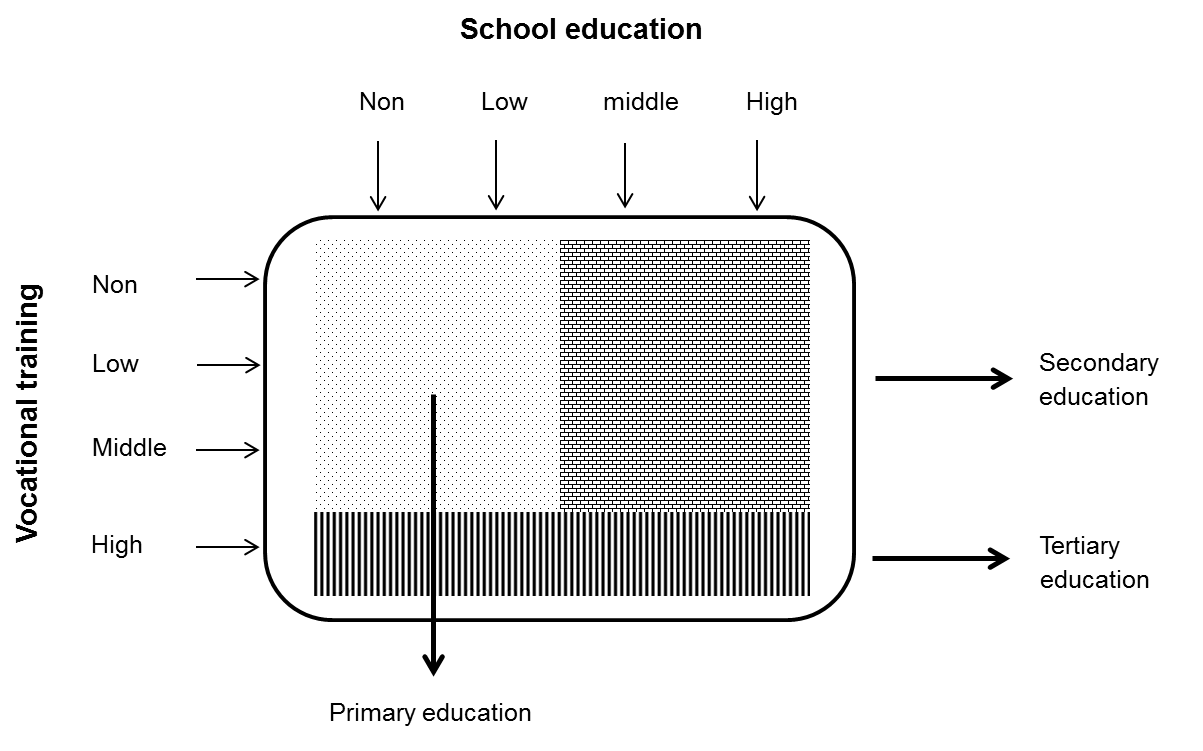


**Figure 1. Level of education assessment, according to Schroedter, Lechert and Lüttinger 2006 (1)**

**References**

(1) Schroedter JH, Lechert Y, Lüttinger P. Die Umsetzung der Bildungsklassifikation CASMIN für die Volkszählung 1970, die Mikrozensus-Zusatzerhebung 1971 und die Mikrozensen 1976-2004 [Transformation of the CASMIN education classification for the census 1970, the micro-census supplement 1971 and the micro-censuses 1976-2004] ZUMA Methodenbericht. 2006;12:1-58.
